# Factors associated with post-traumatic stress disorder in nurses after directly caring for COVID-19 patients: a cross-sectional study

**DOI:** 10.1186/s12912-023-01431-5

**Published:** 2023-08-24

**Authors:** Hyo-Jeong Yoon, Soon Yeung Bae, Jihyun Baek

**Affiliations:** 1 Department of Nursing, Yeungnam University College, Daegu, Republic of Korea; 2 Department of Nursing, Yeungnam University Medical Center, Daegu, Republic of Korea; 3 College of Nursing, Research Institute of Nursing Science, Jeonbuk National University, 567 Baekje-daero, Deokjin-gu, Jeonju-si, Jeollabuk-do 54896 Republic of Korea

**Keywords:** Stress Disorders, Post-traumatic, COVID-19, Nurses, Working Conditions, Organizational Culture, Leadership

## Abstract

**Background:**

Nurses are particularly at risk of suffering from post-traumatic stress disorder (PTSD) owing to their overwhelming workload, risk of infection, and lack of knowledge about the coronavirus disease 2019 (COVID-19). PTSD negatively affects an individual’s health, work performance, and patient safety. This study aims to assess factors related to PTSD among nurses after providing direct care to COVID-19 patients.

**Methods:**

This study is a secondary analysis aimed at identifying factors influencing PTSD among nurses who provided direct care to COVID-19 patients. Data from 168 nurses, collected between October and November 2020, were analyzed. The independent variables were personal, interpersonal, and organizational and COVID-19-related factors (experience of quarantine and direct care of COVID-19 patients), and the dependent variables were PTSD symptoms evaluated based on the PTSD Checklist-5. The nurses’ experience of direct care for COVID-19 patients in the designated COVID-19 isolation wards during the first wave of the pandemic (February 2020 to May 2020) was included.

**Results:**

Among the nurses, 18.5% exhibited symptoms of PTSD. When providing direct care to a patient in the designated COVID-19 isolation ward, nurses witnessing the death of a patient (*p* = .001), low level of nurse staffing (*p* = .008), and inconvenience of electronic health records programs (*p* = .034) were associated with PTSD symptoms. The experience of quarantine owing to COVID-19 was also associated with PTSD symptoms (*p* = .034). Additionally, the higher the nurse managers’ ability, leadership, and support of nurses in the current ward, the higher the possibility of lowering nurses’ PTSD symptoms (*p* = .006).

**Conclusions:**

Governments and hospitals should prepare and implement organizational intervention programs to improve nurse managers’ leadership, nurse staffing levels, and electronic health records programs. Additionally, because nurses who have witnessed the death of a COVID-19 patient or are self-isolating are vulnerable to PTSD, psychological support should be provided.

## Background

Coronavirus disease 2019 (COVID-19) was first detected among patients with pneumonia in December 2019 in Wuhan, China [1]. Since then, COVID-19 has spread rapidly to many countries and regions [2]. South Korea’s first large outbreak in February 2020 occurred in a church in Daegu [3]. To handle the surging demand for hospital care at the onset of the outbreak, the government recruited additional healthcare workers [3]. Research shows that healthcare workers may experience psychological stress because of their overwhelming workload, insufficient psychological preparation, and lack of understanding of COVID-19 in the early stages [4].

Post-traumatic stress disorder (PTSD) is a psychiatric disorder that can affect people who have experienced or witnessed a traumatic event [5]. Healthcare workers are especially vulnerable to PTSD owing to highly stressful work-related situations, such as witnessing death, trauma, and working overtime and in overcrowded settings [6, 7]. Frontline healthcare workers may experience feelings of trauma resulting from fear of infection, shortage of self-protection equipment, heavy work overload, and lack of knowledge about COVID-19 [8, 9]. PTSD rates of 36.5% have been reported for healthcare workers who directly care for COVID-19 patients, and 27.3% for those providing care indirectly [10]. Nurses are more closely connected to patients and face traumatic situations [11]. PTSD is more prevalent in nurses than in other healthcare workers [4, 10, 12]. PTSD may significantly affect mental, emotional, and physical health [13]. Additionally, PTSD is associated with increased turnover intention and diminished concentration and cognitive ability, resulting in medication errors, and disrupts work performance, affecting patient safety and the healthcare organization [14–16]. There is a need to develop a strategy to mitigate the harmful effect of PTSD and promote nurses’ well-being and quality of patient care [16].

Predictors of PTSD is important for identifying those who may be at risk of developing PTSD. Organizational, interpersonal, and intrapersonal factors influence PTSD among nurses [13]. A previous systematic review investigated the risk factors related to PTSD involved in coronavirus outbreaks of severe acute respiratory syndrome (SARS), middle east respiratory syndrome (MERS), and COVID-19 [6]. Some variables were risk and resilience factors, including age, gender, marital status, working role, years of work experience, exposure level, quarantine, social and work support, job organization, and coping styles [6]. Previous studies show that working in COVID-19 units, inadequate nurse staffing [17], and nurse manager leadership [18] were related to higher PTSD among hospital nurses during the COVID‑19 outbreak.

Infectious disease pandemics are expected to cause anticipated worry and PTSD after outbreaks [7, 19]. Considering potential post-COVID-19 issues, it is necessary to establish management strategies for PTSD among nurses after having directly dealt with COVID-19 patients. The spread of COVID-19 was identified in Daegu in February 2020 and hospitals were designated as COVID-19 isolation wards for COVID-19 patients. As the caseload in Daegu decreased in May 2020, some hospitals ceased to operate as COVID-19 isolation wards and began accepting only non-COVID-19 patients. COVID-19 patients were transferred to another designated hospital [20]. Since nurses working in non-COVID-19 hospitals can also be in a post-traumatic situation after providing direct care to COVID-19 patients, they can be examined to identify PTSD-related factors in the post-COVID period.

There is a critical need to develop strategies that address PTSD and promote the well-being of nurses, ensuring the delivery of high-quality patient care. Understanding the predictors of PTSD is essential for identifying at-risk individuals. Given the potential effects of the pandemic, it is crucial to establish management strategies for PTSD among nurses who have provided direct care to COVID-19 patients. Examining the factors related to PTSD in nurses working in non-COVID-19 hospitals after their direct involvement with COVID-19 patients can provide valuable insights into the post-COVID period. This study aims to investigate factors related to PTSD among nurses who provided direct care to COVID-19 patients. Based on an integrative review of PTSD among nurses, we included interpersonal, intrapersonal, and organizational [13], as well as COVID-19 related factors.

## Methods

### Study design

This study is a secondary analysis that aims to identify the factors affecting PTSD among nurses who provided direct care to COVID-19 patients. Data from a previous study [18] was analyzed for this purpose.

### Participants

This study conducted a secondary analysis of data from Bae et al.’s study [18]. Since February 2020, before the WHO declared a COVID-19 pandemic, Daegu has designated dedicated COVID-19 isolation wards. Due to a decrease in the number of COVID-19 patients, three of five private tertiary hospitals ended their operations of dedicated COVID-19 isolation wards in May 2020. Bae et al. [18] conducted the survey five months after three private tertiary hospitals terminated operating dedicated COVID-19 wards.

In this study, 168 nurses, who worked in a dedicated COVID-19 ward and were among the participants of the study by Bae et al. [18]. Bae et al. [18] collected data from October to November 2020 through an online survey while cooperating with nursing departments of three tertiary hospitals in Daegu. The inclusion criterion for participants was nurses working in general wards and integrated nursing care wards, had worked for more than one year and agreed to participate in this study. They had sent an online survey link to a total of 1,500 nurses across three tertiary hospitals. Out of a total of 1,500 nurses, 382 completed the survey (the response rate: 25.5%). In this study, 168 nurses, who worked in a dedicated COVID-19 ward among the participants of the study by Bae et al. [18], were analyzed for the final analysis. Written informed consent was obtained from all participants. With the assistance of the G*Power analysis software, we determined the appropriate sample size for our multiple linear regression study. By considering an effect size of 0.15, power of 0.80, alpha level of 0.05, and 17 explanatory variables, we found that a sample size of 146 was necessary. Consequently, we concluded that the selected sample size for our study was sufficient.

### Measures

#### Intrapersonal and interpersonal characteristics

Intrapersonal and interpersonal characteristics included age, work experience, gender, marital status, cohabitation status, and educational level.

#### Organizational characteristics

The nursing work environment, an organizational characteristic, referred to the ward where nurses worked when the survey was conducted from October to November 2020, and was not a characteristic of designated COVID-19 isolation wards whose operations ended in May 2020.

Organizational characteristics were evaluated using the Korean version of the Practice Environment Scale of Nursing Work Index (PES-NWI) [21], which is a translation of the Nursing Work Index [22]. PES-NWI measures a total of 29 items in five subscales: nurses’ participation in hospital work (nine items), nursing basis for quality of care (nine items), competence, leadership, and nurse’s support of nurse managers (four items), adequacy of manpower and resources (four items), and college nurse–physician relationship (three items). Each item is rated on a 4-point Likert scale (1 = “not at all” to 4 = “absolutely”). The higher the average score of the subscales, the better. At the time of the tool development, the Cronbach’s alpha values of the subscales were 0.71–0.84 [22], and the Cronbach’s alpha values of the Korean tools were 0.80–0.84 [21].

#### COVID-19-related characteristics

COVID-19-related experiences included quarantine and working in a designated COVID-19 isolation ward. Included in the latter were training and orientation of infection control, level of nurse staffing, availability of personal protective equipment (PPE), convenience of electronic health records (EHR), experience of witnessing COVID-19 patient death, and the length of working period in the COVID-19 isolation ward.

#### PTSD

PTSD was evaluated using the PTSD Checklist-5 (PCL-5) [23], which Park [24] translated into Korean. The PCL-5 is a measurement tool designed to meet the PTSD diagnosis criteria of the Diagnostic and Statistical Manual of Mental Disorders, fifth edition (DSM-5). The PCL-5 comprises four subscales (a total of 20 items) namely aggression (five items), avoidance (two items), negative changes in perception and emotion (seven items), and irritability (six items). Each item is rated on a 5-point Likert scale (0 points for “not at all” to 4 points for “very much so”), for a total score of 0 to 80 points. The cutoff point was 33 points; a higher score corresponded to the PTSD diagnostic criteria [25]. The Cronbach’s alpha value of the original tool was 0.94 [26], while the Cronbach’s alpha value of the Korean PCL-5 was 0.91–0.93 [27].

### Data collection

This study was approved by the ethics committee of the university affiliated to the first author and all procedures were conducted per the ethical standards of the 1964 Declaration of Helsinki. All data were computerized to avoid subject identification.

### Statistical analyses

Descriptive statistics were used to examine the intrapersonal, interpersonal, organizational, and COVID-19-related characteristics of nurses. According to the variable characteristics, the relationship between the subject’s characteristics and PTSD was analyzed using the t-test and Pearson’s test. Regression analysis was performed to analyze the factors affecting PTSD. The analysis was performed using SAS 9.4 version (SAS Institute, Cary, NC, USA).

## Results

### Intrapersonal, interpersonal, and organizational characteristics

The average age of the nurses was 31.47 ± 9.25 years and the median age was 28 years. Their mean work experience was 8.91 ± 9.53 years and median was four years. Most participants were women (96.4%), unmarried (70.2%), living with family (85.7%), and had a bachelor’s degree in nursing or higher (85.1%) (Table 1).

**Table 1:** Characteristics and PTSD (N = 168)

	*n* (%) or Mean ± SD
Intrapersonal and Interpersonal characteristics	
Age (years)	31.47 ± 9.25
Below the median (< 28)	80 (47.6)
Above the median (≥ 28)	88 (52.4)
Work experience (years)	8.91 ± 9.53
Below the median (< 4)	83 (49.4)
Above the median (≥ 4)	85 (50.6)
Gender	
Male	6 (3.6)
Female	162 (96.4)
Marital status	
Unmarried	118 (70.2)
Married	50 (29.8)
Cohabitation status	
Living alone	24 (14.3)
Living with family	144 (85.7)
Education level	
Associate’s degree or lower	25 (14.9)
Bachelor’s degree or higher	143 (85.1)
**Organizational characteristic**	
Nursing work environments	2.47 ± 0.40
Nurse participation in hospital affairs	2.33 ± 0.47
Nursing foundations for quality of care	2.68 ± 0.41
Nurse managers’ ability, leadership, and support of nurses	2.69 ± 0.54
Staffing and resource adequacy	2.24 ± 0.62
Collegial nurse-physician relations	2.27 ± 0.61
**COVID-19-related characteristic**	
Experience of quarantine	
No	123 (73.2)
Yes	45 (26.8)
Training/orientation of infection control	
No	82 (48.8)
Yes	86 (51.2)
Level of nurse staffing	
Appropriate	70 (41.7)
Inappropriate	98 (58.3)
Availability of PPE	
Appropriate	78 (46.4)
Inappropriate	90 (53.6)
Convenience of EHR	
Convenience	110 (65.5)
Inconvenience	58 (34.5)
Experience of witnessing COVID-19 patient death	
No	124 (73.8)
Yes	44 (26.2)
Length of working period in the COVID-19 isolation ward	26.61 ± 18.31
PTSD	15.77 ± 16.57
≤ 33	137 (81.5)
> 33	31 (18.5)

Note: COVID-19 = Coronavirus disease 2019; EHR = Electronic health records; PPE = Personal protective equipment; PTSD = Post-traumatic stress disorder; SD = Standard deviation

The overall average PES-NWI was 2.47 ± 0.40. Regarding the average of the five subcategories, nurse managers’ ability, leadership, and support of nurses scored the highest with 2.69 ± 0.54, followed by nursing foundations for quality of care at 2.68 ± 0.41, and nurse participation in hospital affairs at 2.33 ± 0.47; collegial nurse-physician relations scored 2.27 ± 0.61, and staffing and resource adequacy scored the lowest with 2.24 ± 0.62.

### COVID-19-related characteristics

A proportion of 26.8% of the nurses underwent quarantine owing to COVID-19. The experiences of nurses in the designated COVID-19 isolation ward operating from February to May 2020 were as follows: more than half of all nurses underwent training or orientation of infection control (51.2%) and recognized that the level of nurse staffing (58.3%) as well as availability of PPE (53.6%) was inappropriate. One-third of all the nurses (65.5%) stated that EHR was convenient, and 26.2% stated that the COVID-19 patient they cared for died. The average length of days the nurses worked in the designated COVID-19 isolation ward was 26.61 ± 18.31.

### Difference in PTSD by characteristics

Among the nurses, 18.5% exhibited PTSD symptoms, with a PCL-5 score of 34 points or more (Table 1). The t-test showed that nurses’ PTSD- and COVID-19-related characteristics were statistically significant (Table 2). Nurses who perceived an inappropriate level of nurse staffing in the designated COVID-19 isolation ward (*p* = .006) or the EHR as inconvenient (*p* = .021) or witnessed COVID-19 patient death (*p* = .003) exhibited statistically significantly higher PTSD than nurses who did not. Nurse managers’ ability, leadership, and support of nurses, all of which are subscales of the nursing work environment, and PTSD, were statistically significantly correlated (*p* = .023) (Table 3).

**Table 2:** Difference in PTSD by characteristics (N = 168)

	Mean ± SD	*t*	*p*
Intrapersonal and Interpersonal characteristics			
Age (years)			
Below the median (< 28)	13.65 ± 16.40	-1.59	.114
Above the median (≥ 28)	17.69 ± 16.58		
Work experience (years)			
Below the median (< 4)	14.98 ± 17.42	-0.61	.542
Above the median (≥ 4)	16.54 ± 15.75		
Gender			
Male	4.33 ± 7.76	-1.73	.085
Female	16.19 ± 16.67		
Marital status			
Unmarried	14.39 ± 15.91	-1.66	.098
Married	19.02 ± 17.76		
Cohabitation status			
Living alone	15.54 ± 17.51	-0.07	.943
Living with family	15.81 ± 16.47		
Education level			
Associate’s degree or lower	11.92 ± 15.54	-1.26	.209
Bachelor’s degree or higher	16.44 ± 16.70		
**COVID-19-related characteristic**			
Experience of quarantine			
No	14.37 ± 15.83	-1.83	.070
Yes	19.60 ± 18.07		
Training/orientation of infection control			
No	14.93 ± 16.08	-0.64	.522
Yes	16.57 ± 17.08		
Level of nurse staffing			
Appropriate	11.64 ± 14.14	2.78	.006
Inappropriate	18.71 ± 17.59		
Availability of PPE			
Appropriate	13.88 ± 16.61	1.38	.171
Inappropriate	17.40 ± 16.45		
Convenience of EHR			
Convenience	13.63 ± 15.21	2.34	.021
Inconvenience	19.83 ± 18.33		
Experience of witnessing COVID-19 patient death			
No	13.22 ± 14.94	-3.10	.003
Yes	22.95 ± 18.87		
Length of working period in the COVID-19 isolation ward			
Below the median	16.50 ± 17.90	0.54	.587
Above the median	15.10 ± 15.33		

Note: COVID-19 = Coronavirus disease 2019; EHR = Electronic health records; PPE = Personal protective equipment; PTSD = Post-traumatic stress disorder; SD = Standard deviation

**Table 3:** Correlations between PTSD and nursing work environments (N = 168)

	r (*p*)
Nursing work environments	-0.131
	(.092)
Nurse participation in hospital affairs	-0.075
	(.332)
Nursing foundations for quality of care	-0.110
	(.156)
Nurse managers’ ability, leadership, and support of nurses	-0.176
	(.023)
Staffing and resource adequacy	-0.120
	(.120)
Collegial nurse-physician relations	-0.066
	(.397)

Note: PTSD = Post-traumatic stress disorder

### Factors predicting PTSD

Before conducting the regression analysis, the basic hypotheses of multicollinearity and autocorrelation were tested. The variance inflation factors ranged from 1.11 to 3.46, indicating multicollinearity. Therefore, we concluded that multicollinearity did not exist in the data [28]. The Durbin–Watson value was 1.94, indicating no autocorrelation.

Nurses who perceived that the nursing manager’s leadership ability and support in the current ward was adequate had a statistically significant lower PTSD score and were found to be the most influential (β = -0.275, *p* = .006). Evidently, experience of working in a dedicated COVID-19 ward significantly influenced PTSD among nurses (Table 4). The nurse whose patient had died was most affected (β = 0.246, *p* = .001), followed by those who believed that the nurse staffing level was inappropriate (β = 0.205, *p* = .008), and the nurses who experienced discomfort with operating computer programs (β = 0.162, *p* = .034). Additionally, nurses who had undergone quarantine were also found to be significant affected (β = 0.167, *p* = .034).

**Table 4:** Factors related to PTSD (*N* = 168)

	B	SE	β	t	*p*
Intrapersonal and Interpersonal characteristics					
Years of experience	0.262	0.227	0.151	1.153	.251
Female	9.163	6.567	0.103	1.395	.165
Married	1.568	4.628	0.043	0.339	.735
Living with family	-0.373	3.505	-0.008	-0.106	.915
Bachelor’s degree or higher	2.657	3.463	0.057	0.767	.444
Organizational characteristic					
Nurse participation in hospital affairs	3.990	4.215	0.113	0.947	.345
Nursing foundations for quality of care	2.378	4.700	0.059	0.506	.614
Nurse managers’ ability, leadership, and support of nurses	-8.420	2.990	-0.275	-2.816	.006
Staffing and resource adequacy	-1.648	2.399	-0.062	-0.687	.493
Collegial nurse-physician relations	-0.555	2.721	-0.020	-0.204	.839
COVID-19-related characteristic					
Experience of quarantine	6.236	2.906	0.167	2.146	.034
Training/orientation of infection control	0.679	2.513	0.021	0.270	.787
Inappropriate of level of nurse staffing	6.881	2.565	0.205	2.682	.008
Inappropriate of availability of PPE	2.407	2.579	0.073	0.933	.352
Inconvenience of EHR	5.638	2.638	0.162	2.137	.034
Experience of witnessing COVID-19 patient death	9.232	2.800	0.246	3.297	.001
Length of working period in the COVID-19 isolation ward	0.042	0.071	0.047	0.589	.557
R^2^	0.262				
Adj R^2^	0.179				
F	3.14				
*p*	< .001				
Durbin-Watson	1.943				

Note: COVID-19 = Coronavirus disease 2019; EHR = Electronic health records; PPE = Personal protective equipment; PTSD = Post-traumatic stress disorder

## Discussion

This study is novel in that it investigates the factors influencing PTSD among nurses after they provide direct care to COVID-19 patients. The participants of this study did not provide direct care to COVID-19 patients for more than five months after providing direct care to them. Our results show that nurse managers’ ability, leadership, and support of nurses, experience of witnessing COVID-19 patients’ deaths, experience of quarantine, level of nurse staffing, and convenience of EHR were all significantly associated with PTSD among nurses after providing direct care to COVID-19 patients.

After providing direct care to a COVID-19 patient, 18% of nurses experienced PTSD for more than five months. A previous study reported that the prevalence of PTSD during COVID-19 accounted for 55% among nurses and 4–72% among healthcare workers [29, 30]. This disparity in the PTSD prevalence is attributable to variability in measurement tools [31] and points, depending on whether it is evaluated several months after the traumatic event [13, 32]. A longitudinal study found that the proportion of nurses with PTSD was lower during the stable periods than during the outbreak periods [32]. A systematic review reported that post-traumatic stress symptoms accounted for 23.4% of healthcare workers in the acute phase but decreased to 11.9% one year after the psychological distress-causing event [33]. Additionally, the prevalence of PTSD among health workers who experienced natural disasters showed a decreasing trend as the follow-up duration increased [34]. In the early stages of the COVID-19 pandemic, nurses experienced uncertainty and limited knowledge about newly emerging infectious diseases. However, over time, the prevalence of PTSD may have declined because nurses perceived that the degree of threat of the disease reduced following the provision of appropriate protective equipment and adequate protection training [32]. Nevertheless, some nurses still suffer from PTSD; therefore, these nurses and their mental health condition must be carefully considered.

The results of this study show that nurse managers’ ability, leadership, and support of nurses in the current ward were most related to PTSD among nurses who had cared for COVID-19 patients. It had a buffering effect on PTSD. Social support from supervisors proved helpful in reducing PTSD among nurses [35, 36]. The workload of nurse managers to manage the supply of appropriate personnel and supplies is overwhelmingly heavy in emergency situations such as the COVID-19 pandemic. Hence, it is recommended that hospitals allocate additional personnel to psychologically support nurses who provide direct care to COVID-19 patients [37]. Additionally, although it is difficult in an emergency, this study found that proper support and leadership for nurses after a traumatic event can lower PTSD. Hence, it is necessary to develop strategies at the organizational level so that nurse managers can improve the ability, leadership, and support of nurses.

Among the nurses in this study, those who witnessed the death of their patients had higher PTSD scores than those who did not. A previous study reported that nurses who cared for COVID-19 patients who died had a higher risk of developing PTSD [17]. Particularly, the death of COVID-19 patients differs from that of the general population. As COVID-19 patients are isolated from their families and pass away alone, nurses experience overwhelming loss, grief, shame, helplessness, and powerlessness following the patients’ lonely death [38, 39]. However, the length of the time spent working in the COVID-19 ward did not appear to influence PTSD symptoms. In other words, the severity of exposure is considered more important than the period of exposure. Most COVID-19 patients complain of cold-like symptoms or require simple oxygen therapy; therefore, the situation of nursing these patients may not have been recognized as a traumatic event. However, while caring for a dying high-risk patient, they experienced the relevance of perceived threats to their health and life [6]. This study identifies quarantine as an independent factor related to PTSD. This is consistent with a previous study [37], in which quarantined nurses recognized their feelings of vulnerability and were found to be at higher risk for PTSD [6]. When the nurses were quarantined, they suspected that they may have contracted COVID-19. Therefore, interventions to prevent PTSD are needed for nurses who have cared for deceased patients or have been quarantined.

Consistent with a previous study [17], this study demonstrates that PTSD was significantly higher for nurses who responded that nurse staffing was poor than for those who responded that nurse staffing was good. When nurse staffing is inadequate, nurses must care for numerous patients and have a high workload. The higher the number of patients, the more the stress the nurse experiences; exposure to this stress is associated with PTSD [17]. Furthermore, nurses who perceived EHR as inconvenient to use exhibited higher PTSD symptoms. As special medical records for patients with newly emerged infectious diseases were not implemented in the originally used EHR, it was not user-friendly or suitable for nurses caring for COVID-19 patients. It is related to PTSD symptoms because it causes psychological distress when work efficiency is low owing to low EHR reliability and low support for cooperation [40]. The availability of appropriate PPE did not appear to be related to PTSD in this study, which is attributable to the fact that the lack of PPE is not a serious issue in Korea. Following the MERS outbreak, the Korean government prepared a prevention system for infectious diseases after learning [41]. In this study, 46% of the participants stated that they felt there was a lack of PPE. However, in a previous study, nearly all nurses (92.4%) reported difficulty accessing PPE [40]. This could be due to the importance of PPE accessibility. However, the effectiveness of PPE is considered more important in preventing transmission. Previous research shows that the perception of low security while using PPE is associated with higher PTSD, but not with a lack of PPE access [42]. We noted that participants working in the COVID-19 ward with poorer staffing and an inaccurate EHR were at higher risk of developing PTSD, highlighting the importance of organizational support for a proper working environment.

Our study has several limitations. As this is a cross-sectional study using subjective questionnaires in some hospitals in Korea, generalization is limited, causality cannot be identified, and recall bias may exist. Additionally, although the survey was conducted among nurses several months after providing direct care to COVID-19 patients, there are limitations in assuming that it is a fully post-COVID-19 situation because the pandemic is ongoing. However, at the time of the survey, the participants were only caring for non-COVID-19 patients in hospitals where COVID-19 patients were not hospitalized. Furthermore, unlike other Korean cities, Daegu did not experience a second wave of COVID-19 after the first wave, when the number of confirmed cases increased rapidly [43].

## Conclusion

We analyzed factors influencing PTSD among nurses who provided direct care to COVID-19 patients after the COVID-19 pandemic. Consequently, we found that nurse managers’ ability, leadership, and support of nurses in the ward after the COVID-19 pandemic significantly influenced PTSD symptoms among nurses. When providing direct care to COVID-19 patients, nurses were more likely to develop PTSD symptoms if the level of nurse staffing was low or if the EHR was inconvenient. Accordingly, hospitals should prepare and implement organizational intervention programs for the leadership of nurse managers, level of nurse staffing, and EHR program. Additionally, if the COVID-19 patient whom the nurse was taking care of died or the nurse who was quarantined was vulnerable to PTSD symptoms, the corresponding nurse should be provided with psychological and psychiatric support. Further research is needed, in particular, to develop interventions to cultivate nurse managers’ ability, leadership, and support of nurses, as well as interventions to support nurses, and to confirm the effectiveness of such interventions.

## Data Availability

The datasets analyzed during the current study are available from the corresponding author on reasonable request.

## Abbreviations

COVID-19: Coronavirus disease 2019
DSM-5: Diagnostic and statistical manual of mental disorders, fifth edition
EHR: Electronic health records
MERS: Middle east respiratory syndrome
PCL-5: Post-traumatic stress disorder checklist-5
PES-NWI: Practice Environment Scale of Nursing Work Index
PPE: Personal protective equipment
PTSD: Post-traumatic stress disorder
SARS: Severe acute respiratory syndrome
