# Correction: Factors associated with post-traumatic stress disorder in nurses after directly caring for COVID-19 patients: a cross-sectional study

**DOI:** 10.1186/s12912-023-01595-0

**Published:** 2024-02-08

**Authors:** Hyo-Jeong Yoon, Soon Yeung Bae, Jihyun Baek

**Affiliations:** 1 Department of Nursing, Yeungnam University College, Daegu, Republic of Korea; 2 Department of Nursing, Yeungnam University Medical Center, Daegu, Daegu, Republic of Korea; 3 College of Nursing, Research Institute of Nursing Science, Jeonbuk National University, Jeonju, Republic of Korea

**Correction: BMC Nurs 22, 282 (2023)**

**https://doi.org/10.1186/s12912-023-01431-5**

Following publication of the original article [1], the authors reported below errors.

1) In the results of the article, ‘recognized that the level of nurse staffing (58.3%) as well as availability of PPE (53.6%) was **adequate**. One-third of all the nurses (65.5%) stated that EHR was **inconvenient**’ should be ‘recognized that the level of nurse staffing (58.3%) as well as availability of PPE (53.6%) was **inappropriate**. One-third of all the nurses (65.5%) stated that EHR was **convenient**’.

  2) In Table 1, there have been inaccuracies in the values attributed to 'Appropriate' and 'Inappropriate' in the 'Level of nurse staffing,' 'Availability of PPE,' and 'Convenience of EHR' variables. Consequently, 'Appropriate' for 'Level of nurse staffing' should be adjusted from '98 (58.3)' to '70 (41.7),' and 'Inappropriate' should be rectified from '70 (41.7)' to '98 (58.3).' Similarly, for the 'Availability of PPE' variable, 'Appropriate' should be amended from '90 (53.6)' to '78 (46.4),' and 'Inappropriate' should be changed from '78 (46.4)' to '90 (53.6).' Furthermore, the 'Convenience of EHR' variable necessitates correction, with 'Convenience' being revised from '58 (34.5)' to '110 (65.5)' and 'Inconvenience' being altered from '110 (65.5)' to '58 (34.5).'

**Table 1:** Characteristics and PTSD (*N* = 168)

	***n*** **(%) or Mean ± SD**
**Intrapersonal and Interpersonal characteristics**	
Age (years)	31.47 ± 9.25
Below the median (< 28)	80 (47.6)
Above the median (≥ 28)	88 (52.4)
Work experience (years)	8.91 ± 9.53
Below the median (< 4)	83 (49.4)
Above the median (≥ 4)	85 (50.6)
Gender	
Male	6 (3.6)
Female	162 (96.4)
Marital status	
Unmarried	118 (70.2)
Married	50 (29.8)
Cohabitation status	
Living alone	24 (14.3)
Living with family	144 (85.7)
Education level	
Associate’s degree or lower	25 (14.9)
Bachelor’s degree or higher	143 (85.1)
**Organizational characteristic**	
Nursing work environments	2.47 ± 0.40
Nurse participation in hospital affairs	2.33 ± 0.47
Nursing foundations for quality of care	2.68 ± 0.41
Nurse managers’ ability, leadership, and support of nurses	2.69 ± 0.54
Staffing and resource adequacy	2.24 ± 0.62
Collegial nurse-physician relations	2.27 ± 0.61
**COVID-19-related characteristic**	
Experience of quarantine	
No	123 (73.2)
Yes	45 (26.8)
Training/orientation of infection control	
No	82 (48.8)
Yes	86 (51.2)
Level of nurse staffing	
Appropriate	70 (41.7)
Inappropriate	98 (58.3)
Availability of PPE	
Appropriate	78 (46.4)
Inappropriate	90 (53.6)
Convenience of EHR	
Convenience	110 (65.5)
Inconvenience	58 (34.5)
Experience of witnessing COVID-19 patient death	
No	124 (73.8)
Yes	44 (26.2)
Length of working period in the COVID-19 isolation ward	26.61 ± 18.31
PTSD	15.77 ± 16.57
≤ 33	137 (81.5)
> 33	31 (18.5)

Note: *COVID-19* Coronavirus disease 2019, *EHR* Electronic health records, *PPE* Personal protective equipment, *PTSD* Post-traumatic stress disorder, *SD* Standard deviation

3) In Table 2, there has been an inadvertent interchange of values between 'Appropriate' and 'Inappropriate' in the 'Level of nurse staffing,' 'Availability of PPE,' and 'Convenience of EHR' variables. Consequently, 'Appropriate' for 'Level of nurse staffing' should be rectified from “18.71 ± 17.59’ to '11.64 ± 14.14', and 'Inappropriate' should be amended from '11.64 ± 14.14' to '18.71 ± 17.59'. Additionally, the 't' value should be revised from '2.78' to '-2.78.'

**Table 2:** Difference in PTSD by characteristics

	**Mean ± SD**	***t***	***p***
**Intrapersonal and Interpersonal characteristics**			
Age (years)			
Below the median (< 28)	13.65 ± 16.40	-1.59	.114
Above the median (≥ 28)	17.69 ± 16.58		
Work experience (years)			
Below the median (< 4)	14.98 ± 17.42	-0.61	.542
Above the median (≥ 4)	16.54 ± 15.75		
Gender			
Male	4.33 ± 7.76	-1.73	.085
Female	16.19 ± 16.67		
Marital status			
Unmarried	14.39 ± 15.91	-1.66	.098
Married	19.02 ± 17.76		
Cohabitation status			
Living alone	15.54 ± 17.51	-0.07	.943
Living with family	15.81 ± 16.47		
Education level			
Associate’s degree or lower	11.92 ± 15.54	-1.26	.209
Bachelor’s degree or higher	16.44 ± 16.70		
**COVID-19-related characteristic**			
Experience of quarantine			
No	14.37 ± 15.83	-1.83	.070
Yes	19.60 ± 18.07		
Training/orientation of infection control			
No	14.93 ± 16.08	-0.64	.522
Yes	16.57 ± 17.08		
Level of nurse staffing			
Appropriate	11.64±14.14	-2.78	.006
Inappropriate	18.71±17.59		
Availability of PPE			
Appropriate	13.88±16.61	-1.38	.171
Inappropriate	17.40±16.45		
Convenience of EHR			
Convenience	13.63±15.21	-2.34	.021
Inconvenience	19.83±18.33		
Experience of witnessing COVID-19 patient death			
No	13.22 ± 14.94	-3.10	.003
Yes	22.95 ± 18.87		
Length of working period in the COVID-19 isolation ward			
Below the median	16.50 ± 17.90	0.54	.587
Above the median	15.10 ± 15.33		

Note: *COVID-19* Coronavirus disease 2019, *EHR* Electronic health records, *PPE* Personal protective equipment, *PTSD* Post-traumatic stress disorder, *SD* Standard deviation

Similarly, for the 'Availability of PPE' variable, 'Appropriate' should be modified from '17.40 ± 16.45' to '13.88 ± 16.61', and 'Inappropriate' should be adjusted from '13.88 ± 16.61' to '17.40 ± 16.45' The 't' value should also be changed from '1.38' to '-1.38.'

Furthermore, the 'Convenience of EHR' variable necessitates correction, with 'Convenience' being revised from '19.83 ± 18.33' to '13.63 ± 15.21', and 'Inconvenience' should be altered from '13.63 ± 15.21' to '19.83 ± 18.33'. Additionally, the 't' value should be corrected from '2.34' to '-2.34.'

The original article [1] has been corrected.
